# Neuronal Nitric Oxide Synthase Contributes to PTZ Kindling Epilepsy-Induced Hippocampal Endoplasmic Reticulum Stress and Oxidative Damage

**DOI:** 10.3389/fncel.2017.00377

**Published:** 2017-11-28

**Authors:** Xinjian Zhu, Jingde Dong, Bing Han, Rongrong Huang, Aifeng Zhang, Zhengrong Xia, Huanhuan Chang, Jie Chao, Honghong Yao

**Affiliations:** ^1^Department of Pharmacology, Medical School, Southeast University, Nanjing, China; ^2^Department of Geriatric Neurology, Nanjing Brain Hospital, Nanjing Medical University, Nanjing, China; ^3^Department of Pathology, Medical School, Southeast University, Nanjing, China; ^4^Analysis and Test Center, Nanjing Medical University, Nanjing, China; ^5^Nanjing Biomedical Research Institute, Nanjing University, Nanjing, China; ^6^Department of Physiology, Medical School, Southeast University, Nanjing, China

**Keywords:** neuronal nitric oxide synthase, epilepsy, oxidative damage, ER stress, peroxynitrite

## Abstract

Epilepsy is one of the most common chronic neurological disorders which provoke progressive neuronal degeneration. Endoplasmic reticulum (ER) stress has recently been recognized as pivotal etiological factors contributing to epilepsy-induced neuronal damage. However, the specific contribution of epilepsy made to ER stress remains largely elusive. Here we use pentylenetetrazole (PTZ) kindling, a chronic epilepsy model, to identify neuronal nitric oxide synthase (nNOS) as a signaling molecule triggering PTZ kindling epilepsy-induced ER stress and oxidative damage. By genetic deletion of nNOS gene, we further demonstrated that nNOS acts through peroxynitrite, an important member of reactive nitrogen species, to trigger hippocampal ER stress and oxidative damage in the PTZ-kindled mice. Our findings thus define a specific mechanism for chronic epilepsy-induced ER stress and oxidative damage, and identify a potential therapeutic target for neuroprotection in chronic epilepsy patients.

## Introduction

Neuronal nitric oxide synthase (nNOS) is widely expressed in neurons of the brain, where it produces nitric oxide (NO) during the conversion of L-arginine to citrulline, with the participation of NADPH. nNOS is structurally coupled with NMDA receptor by a scaffolding protein (PSD-95), activation of nNOS depends on its association with PSD-95 and NMDA receptor-mediated calcium influx (Sattler et al., 1999). Our previous study has demonstrated that PTZ kindling activated NMDA receptor (Zhu et al., 2015) and induced nNOS activity (Zhu et al., 2016a), suggesting nNOS is stimulated through NMDA receptor activation in the epileptic condition.

Excitotoxicity has been considered to be a major pathological process of neuronal death in chronic epilepsy (Liu et al., 2009). A growing body of evidence suggests that oxidative stress has been implicated in the mechanism of excitotoxicity and neurodegeneration in the development of epilepsy (Rowley and Patel, 2013; Mishra et al., 2015). It is reported that loss of interneurons in the hippocampus of epileptic brain disrupts the balance of excitation and inhibition and causes hyperexcitability consequently (Hofmann et al., 2016). This neuronal hyperexcitability may result in neurodegeneration by generation of excessive reactive oxygen/nitrogen species and by impairing the endogenous antioxidant system.

ER stress is a condition where proper ER structure and functioning were disrupted. ER stress has been suggested to be involved in various neurological diseases including epilepsy (Yamamoto et al., 2006; Ko et al., 2015). A growing body of evidence suggests that prolonged ER stress triggers intracellular signals leading to neurodegeneration in epileptic brain (Sokka et al., 2007; Ko et al., 2015), indicating ER stress plays an important role in epilepsy-induced neurodegeneration. Thus, a better understanding of the mechanism of seizure or epilepsy-induced ER stress is crucial to uncover the potential link between ER stress and epilepsy.

Pentylenetetrazole (PTZ) kindling is a chronic epilepsy model which is characterized by sustained increase in seizure susceptibility. PTZ kindling causes alterations in the molecular and cellular levels, which are responsible for the oxidative stress and neurodegenerative changes in the hippocampus. In our previous study, we observed a significant higher level of nNOS expression and its enzymatic activity in the hippocampus of PTZ-kindled mice (Zhu et al., 2016a). Here, in this study, we sought to further determine whether nNOS is involved in PTZ kindling-induced ER stress and oxidative damage.

## Materials and methods

### Animals

Male C57BL/6J mice (4–6 weeks old; weighing 19 ± 2 g) were purchased from Nanjing Biomedical Research Institute of Nanjing University (NBRI) (Nanjing, China). Mice lacking nNOS (B6; 129S4-Nos1^tm1Plh^) obtained from NBRI were backcrossed to C57BL/6J strain and the heterozygotes were intercrossed to obtain mutation homozygotes. Male homozygous nNOS-null (nNOS^−/−^) and their wild-type (nNOS^+/+^) littermates (4–6 weeks old) were used in the experiment. The animals were maintained in a 12 h light/dark cycle and allowed free access to food and water. Drug intraperitoneally injection and stereotaxic surgery on animals may cause painful experiences, anxiety and depression. Pain, suffering or distress is minimized to the greatest possible extent, but without affecting the results of the experiment. Stereotaxic surgery is performed after adequate anesthesia and postoperative care is implemented to reduce suffering after the surgery. All animal related experiments were performed under the protocol approved by the Animal Care and Use Committee of Medical School of Southeast University.

### Drugs

Pentylenetetrazole (PTZ, 35 mg/kg), 7-nitroindazole (7-NI, 30 mg/kg), dizocilpine hydrogen maleate (MK-801, 0.1 mg/kg) were intraperitoneally injected. Stereotaxic surgery was used to bilaterally deliver (3-morpholino-sydnonimine, SIN-1) (250 nM) and [5,10,15,20-Tetrakis-(4-sulfonatophenyl) porphyrinato iron (III), chloride, FeTPPS] (100 nM) into the hippocampus. The stereotaxic coordinates used were 2.3 mm posterior to the bregma, 2.2 mm below dura, and 1.5 mm lateral to the midline. A total of 1 μl volume was delivered over 2 min to allow for full diffusion of the injected solution. PTZ, 7-NI, MK-801, and SIN-1 were purchased from Sigma Aldrich (St. Louis, MO, USA). FeTPPS was purchased from Santa Cruz (Dallas, TX, USA).

### PTZ kindling procedure

PTZ kindling model was induced as we previously described (Zhu et al., 2015). Briefly, mice were intraperitoneally injected with subconvulsive dose of PTZ (35 mg/kg) every 24 h for 11 total injections. Vehicle control mice received the same number of saline injections. After each injection, seizure behavior was video monitored for 30 min. The intensity of seizure was evaluated using the following scale. Stage 0, no response; Stage 1, ear and facial twitching; Stage 2, convulsive twitching axially through the body; Stage 3, myoclonic jerks and rearing; Stage 4, wild running and jumping; Stage 5, generalized tonic-clonic seizures; and Stage 6, death (Schroder et al., 1993; Becker et al., 1995; Mizoguchi et al., 2011).

### Brain tissue processing

For western blot, the mice were sacrificed and the hippocampus was immediately dissected and was snap-frozen and stored at −80°C until assayed. For immunocytochemistry, the mice were deeply anesthetized and were transcardially perfused with 100 mL of cold saline, followed by 50 mL of 4% paraformaldehyde (PFA). The mouse brains were post-fixed overnight at 4°C, and then transferred to 30% sucrose i. Serial coronal sections (25 μm) throughout the hippocampus were cut using a cryostat (Leica Microsystems, Wetzlar, Germany). Every sixth section throughout the hippocampus were collected for immunocytochemistry studies as we described previously (Zhu et al., 2016b). For transmission electron microscopy (TEM) assay, mice were underwent anesthesia and transcardial perfusion with 4% PFA and 2% glutaraldehyde. The brains were then removed and placed in 2% glutaraldehyde until assayed.

### NOS activity assay and no content measurement

NOS activity was determined as we previously described (Zhu et al., 2016a). In brief, the hippocampus was homogenized and centrifuged at 10,000 g for 20 min at 4°C. The supernatant was ultracentrifuged at 100,000 g for 15 min at 4°C using a 300 kDa molecular weight cut-off filter (Spectrum laboratories Inc.) and the protein concentration was measured by using a BCA protein assay kit (Pierce, Rockford, IL, USA). nNOS activity in the filtrates were measured by using a nNOS assay kit (Jiancheng Bioengineering Co., Nanjing, China). nNOS activities was expressed as unit (U). Hippocampal NO production were detected as we previously described (Zhu et al., 2016a). Briefly, hippocampal tissues were homogenized and the supernatants were used to measure the NO concentration by using a NO assay kit (Jiancheng Bioengineering Co., Nanjing, China). NO concentration was expressed as nmol/mg protein.

### Measurement of superoxide anion production

Superoxide anion (${\text{O}}_{2}^{-}$) production was detected using a LumiMax Superoxide Anion Detection kit (Agilent Technologies, La Jolla, CA, USA) following the manufacture's protocol. Briefly, 50 μg sample proteins were suspended in 100 μl assay medium, and then mixed with 100 μl of reagent mixture containing 0.2 mM luminol and 0.25 mM enhancer. Light emissions were measured by a luminometer. ${\text{O}}_{2}^{-}$ content was expressed as relative light units (RLU)/μg protein/min.

### Measurement enzymatic activities of SOD, CAT, and GSH-Px

Enzymatic activities of superoxide dismutase (SOD), catalase (CAT), and glutathione peroxidase (GSH-Px) were measured as we described previously (Zhu et al., 2015), the dissected hippocampal tissues were homogenized in tissue lysis buffer (Beyotime Biotechnology, China). The homogenates were then centrifuged at 3,000 rpm for 15 min. The protein concentration was determined using a BCA protein assay kit (Pierce, Rockford, IL, USA). The enzymatic activities of SOD, CAT and GSH-Px were then measured by using commercially available kits (Jiancheng Bioengineering, Nanjing China). The activities of SOD, CAT, and GSH-Px were expressed as units per mg protein.

### Measurement of lipid peroxidation and protein oxidation

Malondialdehyde (MDA) and 4-hydroxy-2-nonenal (4-HNE) were used as common indicators to measure lipid peroxidation. MDA and 4-HNE were detected by MDA assay kit (Jiancheng Bioengineering, Nanjing China) and 4-HNE assay kit (Cell Biolabs, San Diego, CA), respectively. The protein oxidation was determined by measuring protein carbonyl using a protein carbonyl content assay kit (Sigma-Aldrich, St. Louis, USA). MDA and protein carbonyl content was expressed as nmol per mg protein while 4-HNE content was expressed as μg per g protein.

### Immunocytochemistry and cell counting

The immunocytochemistry studies were performed as we previously described (Zhu et al., 2016b). Briefly, the sections were incubated in 2 × SSC/formamide at 65°C for 50 min, and then transferred to 2 M HCl at 30°C for 30 min, the sections were then rinsed in 0.1 M boric acid (pH 8.5) for 10 min, incubated in 1% H_2_O_2_ in PBS for 30 min and blocked in 3% normal goat serum containing 0.3% (w/v) Triton X-100 and 0.1% BSA at room temperature for 1 h, followed by incubation with mouse anti-3-nitrotyrosine (3-NT) (1:200; Abcam Temecula, CA, USA, Cat^#^ ab61392); mouse anti-4-hydroxynonenal (4-HNE) (1:200; Abcam Temecula, CA, USA, Cat^#^ab48506); mouse anti-GRP78 (1:200; Santa Cruz, Texas, USA, Cat^#^sc373738) and rabbit anti-CHOP (1:200; Abcam Temecula, CA, USA, Cat^#^ab179823) antibody at 4°C overnight. For DAB staining, the sections were developed with super ABC kit (Boster, Wuhan, China) for 3-NT staining, for immunofluorescence assay, the sections were incubated with a TRITC-conjugated goat anti-mouse antibody (1:200; Cwbiotech, Beijing, China) for 4-HNE and GRP78 labeling and a TRITC-conjugated goat anti-rabbit antibody (1:200; Cwbiotech, Beijing, China) for CHOP labeling respectively. The sections were then rinsed and mounted on gelatin-coated slides in DAPI-anti fade mounting medium (SouthernBiotech, Birmingham, Alabama, USA). The images of 4-HNE, GRP78 and CHOP staining were captured with a confocal laser scanning microscope (Olympus LSM-GB200, Japan). The counting of the 3-NT, 4-HNE, GRP78, and CHOP-positive cells was performed as we previously described (Zhu et al., 2016a). Briefly, one experimenter coded all slides from the experiments before quantitative analysis. Eight visual fields (0.6 mm^2^) of hippocampal CA1 region were selected and photographically captured in every sixth section from a series of hippocampal sections. The numbers of 3-NT, 4-HNE, GRP78, and CHOP positive cells in each selected visual field were counted and quantified by using the Image J software (NIH, Bethesda, MD, USA). The data are presented as the number of cells per visual field.

### Fluoro-jade staining

For Fluoro-Jade staining, sections were washed, mounted on glass slides and dried overnight. The slides were immersed for 3 min in absolute ethanol, for 1 min in 70% ethanol, for 1 min in distilled water, and then transferred to a solution containing 0.01% Fluoro-Jade B (EMD Millipore, Darmstadt, Germany) and 0.1% acetic acid for 30 min on a shaker. After three 10 min washes, the slides were finally cover slipped. Fluoro-Jade-positive cells in CA1 pyramidal cell layer of the hippocampus were counted by the use of Image J software (NIH, Bethesda, MD, USA).

### Western blotting

After homogenization, the hippocampal tissues were lysed and centrifuged at 12,000 rpm for 15 min. The protein concentration was measured by using a BCA protein assay kit (Pierce, Rockford, IL, USA). An equal amount of protein from each sample was applied to 12% acrylamide denaturing gels (SDS-PAGE) and were transferred to nitrocellulose membranes (Amersham, Little Chalfont, UK) by using a Bio-Rad mini-protein-III wet transfer unit (Hercules, CA, USA) overnight at 4°C.

The membranes were then blocked in 5% non-fat milk in TBST (10 mmol/l Tris pH = 7.6, 150 mmol/L NaCl, 0.01%Tween-20) for 1 h at room temperature. Next the membranes were incubated with mouse anti-3-NT (1:2,500; Abcam, Temecula, CA, USA, Cat^#^ ab61392), rabbit anti-CHOP (1:2,000; Abcam, Temecula, CA, USA, Cat^#^ab179823), mouse anti-GRP78 (1:2,500; Santa Cruz, Texas, USA, Cat^#^sc373738) and rabbit anti-β-actin (1:5,000; Sigma-Aldrich, St, Louis, USA, Cat^#^SAB2100037) in TBST overnight at 4°C. The membranes were then washed with TBST and incubated with HRP-linked secondary antibody (Boster Bioengineering, Wuhan, China) diluted 1:5,000 for 1 h. Following several washes with TBST, the binding antibodies were detected with an enhanced chemiluminescence (ECL) reagents (Millipore, Billerica, MA, USA) by using a MicroChemi chemiluminescent image analysis system (DNR Bio-imaging Systems, Jerusalem, Israel). Density of blots was analyzed by the Image J software (NIH, Bethesda, MD, USA).

### Transmission electron microscopy

For TEM assay, mice were transcardially perfused and the hippocampus was removed. Next, the tissue samples of CA1 subfield was dissected out of the hippocampus. Tissue samples were then diced and fixed in 2.5% glutaraldehyde. After that, tissue blocks were trimmed and cut to a thickness of 60–70 nm sections. Ultrathin sections were then collected on 300-mesh coated grids. Images were captured using a transmission electron microscope (JEOL, JEM-1010, Tokyo, Japan). Eight visual fields (800 μm^2^) were randomly selected from each sample for further analysis.

### Statistical analysis

All data are presented as the means ± S.E.M. Statistical significance was determined by using unpaired two-tailed Student's *t*-test for two group's comparison and by using one-way or two-way ANOVA for multi-group comparisons. Tukey's test was used for *post-hoc* comparisons. Differences were considered to be significant for values of *p* < 0.05.

## Results

### PTZ kindling-induced increase of nNOS activity is dependent on NMDA receptor activation

**Figure 1:**
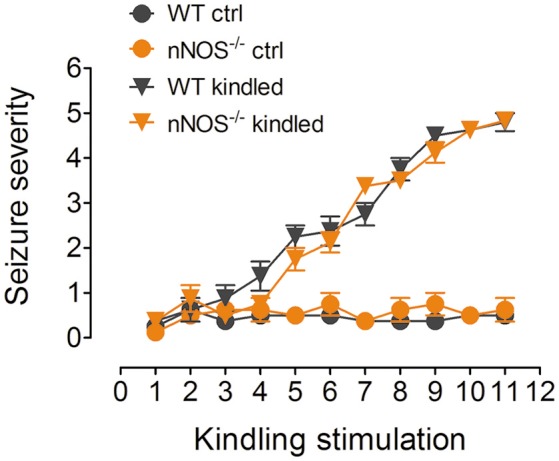
PTZ induces kindling in nNOS^−/−^ mice and their wildtype littermates. Kindling was induced by intraperitoneally injecting the mice with subconvulsive dose of PTZ (35 mg/kg) every other day for 11 total injections. Vehicle control mice received the same number of saline injections. The mice showing more than three consecutive stage 4 seizures were considered to be fully kindled. After repeatedly being treated with PTZ, both of the nNOS^−/−^ mice and their wildtype littermates have achieved progressive increase of seizure severity and finally have been kindled (*n* = 8).

**Figure 2:**
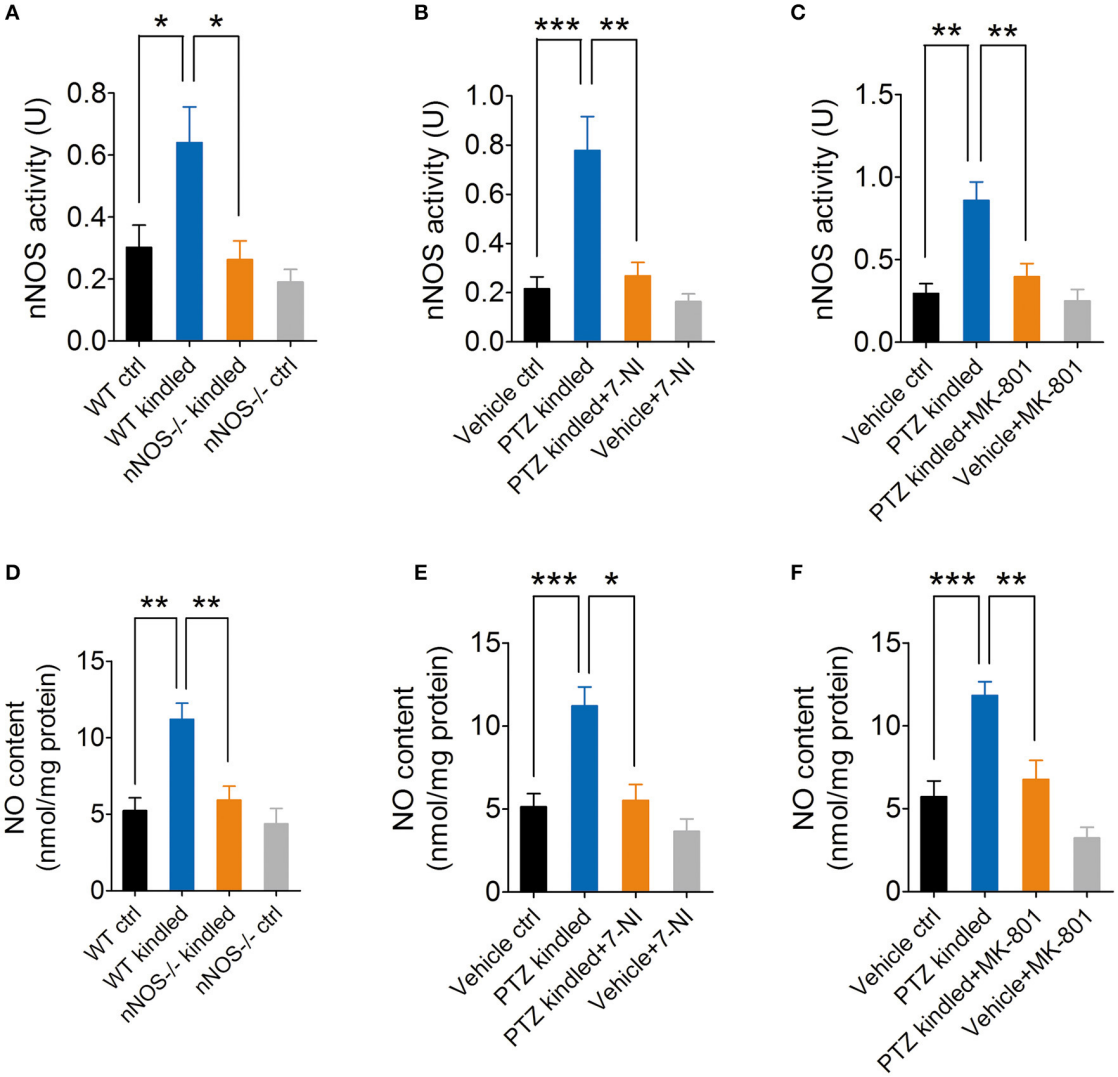
PTZ kindling-induced increase of nNOS activity is dependent on NMDA receptor activation. **(A)** Bar graph showing the enzymatic activities of nNOS in wildtype control (WT ctrl), wildtype kindled (WT kindled), nNOS knock out control (nNOS^−/−^ ctrl) and nNOS knock out kindled (nNOS^−/−^ kindled) mice (*n* = 5). Two-way ANOVA revealed a significant main effect of PTZ treatment [*F*_(1, 16)_ = 7.02, *p* = 0.018], genotype [*F*_(1, 16)_ = 10.02, *p* = 0.006], as well as PTZ treatment × genotype interaction [*F*_(1, 16)_ = 2.95, *p* = 0.015]. A Tukey *post-hoc* test revealed that WT kindled mice had higher level of nNOS activity than WT ctrl mice (*p* = 0.018), and nNOS^−/−^ kindled mice had significant lower level of nNOS activity than WT kindled mice (*p* < 0.016). **(B)** Bar graph showing the enzymatic activities of nNOS in vehicle control (Vehicle ctrl), PTZ kindled (PTZ kindled), PTZ-kindled 7-NI treated (PTZ-kindled+7-NI), and 7-NI treated (Vehicle+7-NI) mice (*n* = 5). Two-way ANOVA revealed a significant main effect of PTZ treatment [*F*_(1, 16)_ = 17.16, *p* < 0.001], 7-NI treatment [*F*_(1, 16)_ = 12.43, *p* = 0.003], as well as PTZ treatment × 7-NI treatment [*F*_(1, 16)_ = 8.26, *p* = 0.011]. A Tukey *post-hoc* test revealed that PTZ-kindled mice had significant higher level of nNOS activity than Vehicle ctrl mice (*p* < 0.001), and PTZ-kindled+7-NI mice had significant lower level of nNOS activity than PTZ-kindled mice (*p* < 0.001). **(C)** Bar graph showing the enzymatic activities of nNOS in vehicle control (Vehicle ctrl), PTZ kindled (PTZ kindled), PTZ-kindled MK-801 treated (PTZ-kindled+MK-801) and MK-801 treated (Vehicle+MK-801) mice (*n* = 5). Two-way ANOVA revealed a significant main effect of PTZ treatment [*F*_(1, 16)_ = 18.16, *p* < 0.001], MK-801 treatment [*F*_(1, 16)_ = 9.35, *p* = 0.008], as well as PTZ treatment × MK-801 treatment [*F*_(1, 16)_ = 6.27, *p* = 0.023]. A Tukey *post-hoc* test revealed that PTZ-kindled mice had significant higher level of nNOS activity than Vehicle ctrl mice (*p* = 0.001), and PTZ-kindled+MK-801 mice had significant lower level of nNOS activity than PTZ-kindled mice (*p* = 0.001). **(D)** Bar graph showing the NO production in WT ctrl, WT kindled, nNOS^−/−^ ctrl, and nNOS^−/−^ kindled mice (*n* = 5). Two-way ANOVA revealed a significant main effect of PTZ treatment [*F*_(1, 16)_ = 15.66, *p* = 0.001], genotype [*F*_(1, 16)_ = 10.43, *p* = 0.005], as well as PTZ treatment × genotype interaction [*F*_(1, 16)_ = 5.38, *p* = 0.034]. A Tukey *post-hoc* test revealed that WT kindled mice had higher level of nNOS activity than WT ctrl mice (*p* = 0.004), and nNOS^−/−^ kindled mice had significant lower level of nNOS activity than WT kindled mice (*p* = 0.006). **(E)** Bar graph showing the NO production in Vehicle ctrl, PTZ kindled, PTZ-kindled+7-NI, and Vehicle+7-NI mice (*n* = 5). Two-way ANOVA revealed a significant main effect of PTZ treatment [*F*_(1, 16)_ = 18.31, *p* < 0.001], 7-NI treatment [*F*_(1, 16)_ = 14.89, *p* = 0.001], as well as PTZ treatment × 7-NI treatment [*F*_(1, 16)_ = 5.16, *p* = 0.037]. A Tukey *post-hoc* test revealed that PTZ-kindled mice had significant higher level of NO production than Vehicle ctrl mice (*p* < 0.001), and PTZ-kindled+7-NI mice had significant lower level of NO production than PTZ-kindled mice (*p* = 0.017). **(F)** Bar graph showing the quantification of NO production in Vehicle ctrl, PTZ kindled, PTZ-kindled+MK-801 and Vehicle+MK-80 mice (*n* = 5). Two-way ANOVA revealed a significant main effect of PTZ treatment [*F*_(1, 16)_ = 27.91, *p* < 0.001], MK-801 treatment [*F*_(1, 16)_ = 17.15, *p* < 0.001], but no significant effect of PTZ treatment × MK-801 treatment [*F*_(1, 16)_ = 1.97, *p* = 0.179]. A Tukey *post-hoc* test revealed that PTZ-kindled mice had significant higher level of NO production than Vehicle ctrl mice (*p* < 0.001), and PTZ-kindled+MK-801 mice had significant lower level of NO production than PTZ-kindled mice (*p* = 0.001). Values are means ± S.E.M. ^*^p < 0.05, ^**^p < 0.01, ^***^p < 0.001, Two-way ANOVA.

Our previous study has demonstrated that hippocampal nNOS mRNA level, protein content and enzymatic activity were significantly increased after the mice were fully kindled (Zhu et al., 2016a). Here in this study, we have used two independent approaches to inhibit nNOS and measure the hippocampal enzymatic activity of nNOS under control and PTZ kindling conditions. In a genetic approach, we used a nNOS deficient mice model (nNOS ^−/−^). Our results show that both of nNOS^−/−^ mice and their wildtype littermates can be successfully kindled by repetitive PTZ treatment (Figure 1). In wildtype mice, hippocampal nNOS enzymatic activity was significantly increased 24 h after they are kindled (Figure 2A). Although nNOS^−/−^ mice can be successfully kindled, the hippocampal enzymatic activity of nNOS in nNOS^−/−^ kindled mice is significantly decreased than that of wildtype kindled mice (Figure 2A). The enzymatic activity of nNOS in nNOS^−/−^ control mice remains similar with those of wildtype control mice (Figure 2A). In a pharmacological approach, we treated mice with nNOS inhibitor 7-NI. Consistently, we find that inhibition of nNOS by 7-NI significantly decreased the nNOS enzymatic activity (Figure 2B). nNOS is structurally coupled with NMDA receptor by PSD-95, raising the possibility that nNOS enzymatic activity is dependent on NMDA receptor activation. To test this hypothesis, we treated mice with a non-competitive NMDA receptor antagonist, MK-801 to block NMDA receptor and measure the nNOS enzymatic activities under control and PTZ kindling conditions. Our results show that blocking NMDA receptor with MK-801 suppressed PTZ kindling-induced increase of nNOS enzymatic activity (Figure 2C), while blocking NMDA receptor in control mice has very limited effects on nNOS enzymatic activity (Figure 2C). In agreement with the result of nNOS enzymatic activity, NO content in the hippocampus of PTZ-kindled mice was significantly increased compared to control mice, while NO content in the hippocampus of nNOS^−/−^ and (Figure 2D) 7-NI-treated (Figure 2E) kindled mice was significantly decreased than that of PTZ-kindled mice (Figure 2D). Furthermore, blocking NMDA receptor with MK-801 suppressed PTZ kindling-induced increase of NO production (Figure 2F). Taken together, these results suggest that the enhancement of nNOS enzymatic activity and NO production induced by PTZ kindling is dependent on NMDA receptor activation and inhibition of nNOS suppresses PTZ kindling-induced nNOS activity and NO production.

### PTZ kindling-induced peroxynitrite formation is dependent on nNOS activity

**Figure 3:**
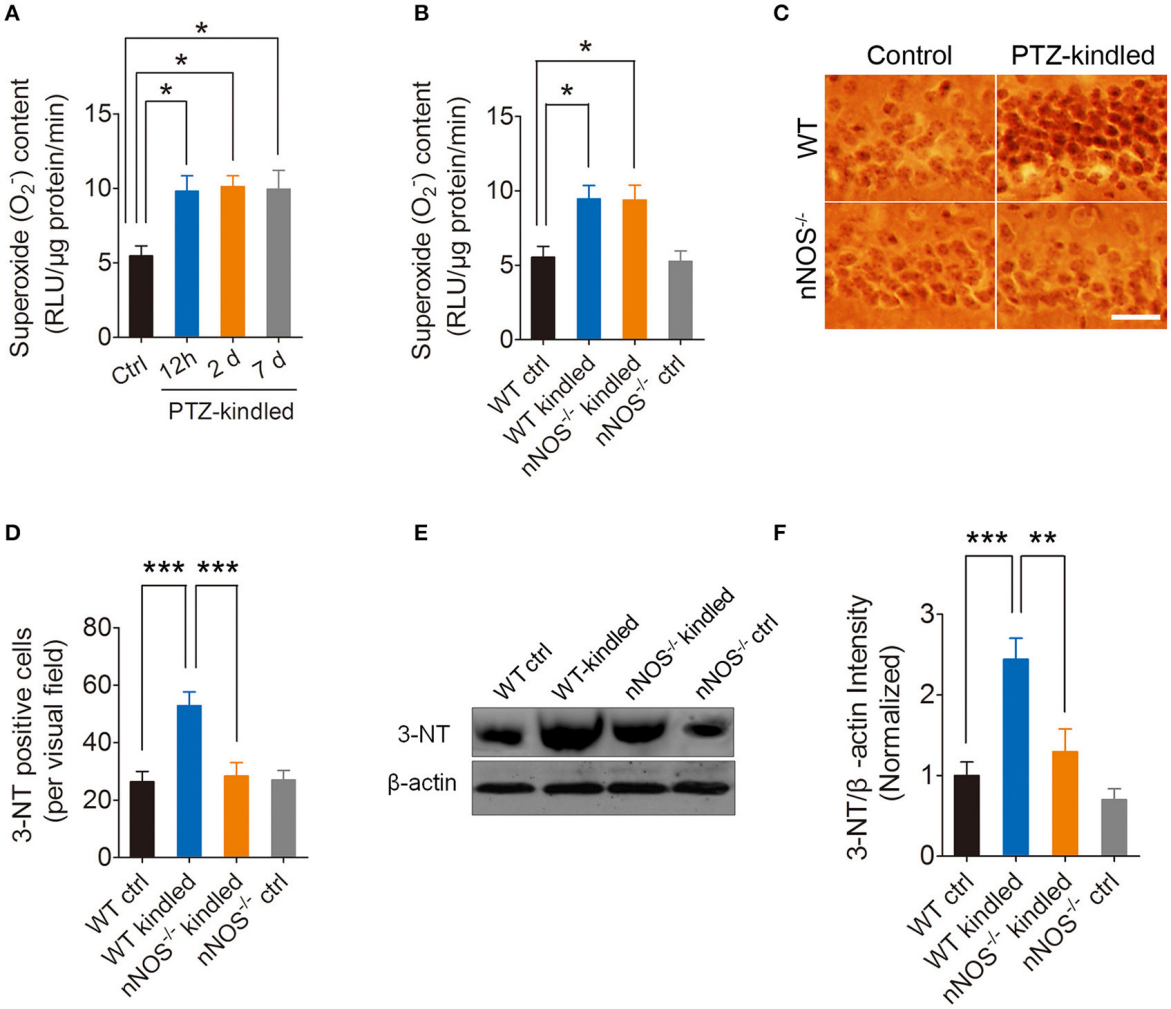
PTZ kindling induces hippocampal superoxide anion (${\text{O}}_{2}^{-}$) production and peroxynitrite (ONOO^−^) formation. **(A)** Bar graph showing the quantification of the ${\text{O}}_{2}^{-}$ content in the hippocampus of control and PTZ-kindled mice (12 h, 2 and 7 days after kindling) [*F*_(1, 16)_ = 5.479, *p* = 0.026, 12 h PTZ-kindled vs. ctrl; *p* = 0.017, 2 d PTZ-kindled vs. ctrl; *p* = 0.021, 7 d PTZ-kindled vs. ctrl, One-way ANOVA] (*n* = 5). **(B)** Bar graph showing the quantification of the ${\text{O}}_{2}^{-}$ content in the hippocampus of WT ctrl, WT kindled, nNOS^−/−^ ctrl and nNOS^−/−^ kindled mice (*n* = 5). Two-way ANOVA revealed a significant main effect of PTZ treatment [*F*_(1, 16)_ = 22.69, *p* < 0.001] but no significant effect of genotype [*F*_(1, 16)_ = 0.05, *p* = 0.835], nor PTZ treatment × genotype interaction [*F*_(1, 16)_ = 0.01, *p* = 0.911]. A Tukey *post-hoc* test revealed that WT kindled mice had higher level of ${\text{O}}_{2}^{-}$ content than WT ctrl mice (*p* = 0.03), and nNOS^−/−^ kindled mice also had significant higher level of ${\text{O}}_{2}^{-}$ content than WT ctrl mice (*p* = 0.01). **(C)** Representative images of 3-nitrotyrosine (3-NT) immunostaining in the hippocampus of WT ctrl, WT kindled, nNOS^−/−^ ctrl and nNOS^−/−^ kindled mice. **(D)** Bar graph showing the quantification of the 3-NT immunopositive cells in the hippocampus of WT ctrl, WT kindled, nNOS^−/−^ ctrl and nNOS^−/−^ kindled mice (*n* = 8). Two-way ANOVA revealed a significant main effect of PTZ treatment [*F*_(1, 28)_ = 11.27, *p* = 0.002] and genotype [*F*_(1, 28)_ = 8.27, *p* = 0.008], as well as PTZ treatment × genotype interaction [*F*_(1, 28)_ = 9.16, *p* = 0.005]. A Tukey *post-hoc* test revealed that WT kindled mice had more 3-NT positive cells than WT ctrl mice (*p* < 0.001), and nNOS^−/−^ kindled mice had fewer 3-NT positive cells than WT kindled mice (*p* < 0.001). **(E,F)** Western blots and quantification of 3-NT protein levels in WT ctrl, WT kindled, nNOS^−/−^ ctrl, and nNOS^−/−^ kindled mice (*n* = 5). Two-way ANOVA revealed a significant main effect of PTZ treatment [*F*_(1, 16)_ = 21.35, *p* < 0.001] and genotype [*F*_(1, 16)_ = 10.76, *p* = 0.005], but no significant effect of PTZ treatment × genotype interaction [*F*_(1, 16)_ = 3.68, *p* = 0.07]. A Tukey *post-hoc* test revealed that WT kindled mice had higher level of 3-NT than WT ctrl mice (*p* < 0.001), and nNOS^−/−^ kindled mice had significant lower level of 3-NT than WT kindled mice (*p* = 0.002). Values are means ± S.E.M. ^*^p < 0.05, ^**^p < 0.01, ^***^p < 0.001. One-way and Two-way ANOVA, Scale bar=50 μm.

Oxidative stress has been shown to occur throughout epilepsy development and is regarded as an important mechanism in the pathogenesis of epilepsy (Liang and Patel, 2004, 2006; Patel, 2004; Waldbaum and Patel, 2010; Ryan et al., 2014). To explore whether PTZ kindling induces oxidative stress, and if so, whether this PTZ kindling-induced oxidative stress is dependent on nNOS signaling, we firstly detected hippocampal superoxide anion (${\text{O}}_{2}^{-}$), which is an important reactive oxygen species (ROS) under normal and PTZ kindling conditions. Our results show that hippocampal ${\text{O}}_{2}^{-}$ production in the wildtype mice started to increase after they were kindled and stabled at a high level up to 7 days post kindling (Figure 3A). To further determine whether PTZ kindling-induced ${\text{O}}_{2}^{-}$ production is dependent on nNOS signaling, we detected ${\text{O}}_{2}^{-}$ production in nNOS^−/−^ mice and their wildtype littermates under normal and PTZ kindling conditions. Our results show that the ${\text{O}}_{2}^{-}$ production in the hippocampus of wildtype and nNOS^−/−^ kindled mice was significantly increased compared to wildtype control mice and there is no significant difference of ${\text{O}}_{2}^{-}$ production between nNOS^−/−^ and wildtype kindled mice (Figure 3B), suggesting PTZ kindling-induced hippocampal ${\text{O}}_{2}^{-}$ production is independent of nNOS activation.

Peroxynitrite is one of the important members of reactive nitrogen species (RNS), causing nitrosative stress. Peroxynitrite formation has been ascribed to the reaction of the free radical superoxide anion with the free radical NO *in vivo* (Pacher et al., 2007; Szabo et al., 2007). Here we measured peroxynitrite by detecting 3-nitrotyrosine (3-NT) expression using immunohistochemistry and western blot. Our immunohistochemistry results showed that hippocampal 3-NT positive cells are significantly increased in wildtype kindled mice compared to wildtype control mice (Figures 3C,D). However, the 3-NT positive cells in the hippocampus of nNOS^−/−^ kindled mice significantly decreased compared to wildtype kindled mice (Figures 3C,D), while the 3-NT positive cells in the hippocampus of nNOS^−/−^ control mice remain similar with those of wildtype control mice (Figures 3C,D). Consistently, our western blot results show that 3-NT protein level in the hippocampus of wildtype kindled mice was significantly higher compared to wildtype control mice (Figures 3E,F). However, 3-NT protein level in the hippocampus of nNOS^−/−^ kindled mice was significantly lower than that of wild type kindled mice (Figures 3E,F), while the protein level of 3-NT between nNOS^−/−^ control and wildtype control mice remains similar (Figures 3E,F), suggesting PTZ kindling-induced hippocampal peroxynitrite production is dependent of nNOS activation. Taken together, these results demonstrated that PTZ kindling induces superoxide and peroxynitrite production in the hippocampus. Moreover, PTZ kindling-induced peroxynitrite formation is dependent on nNOS activity.

### PTZ kindling provokes hippocampal oxidative stress through activation of nNOS

**Figure 4:**
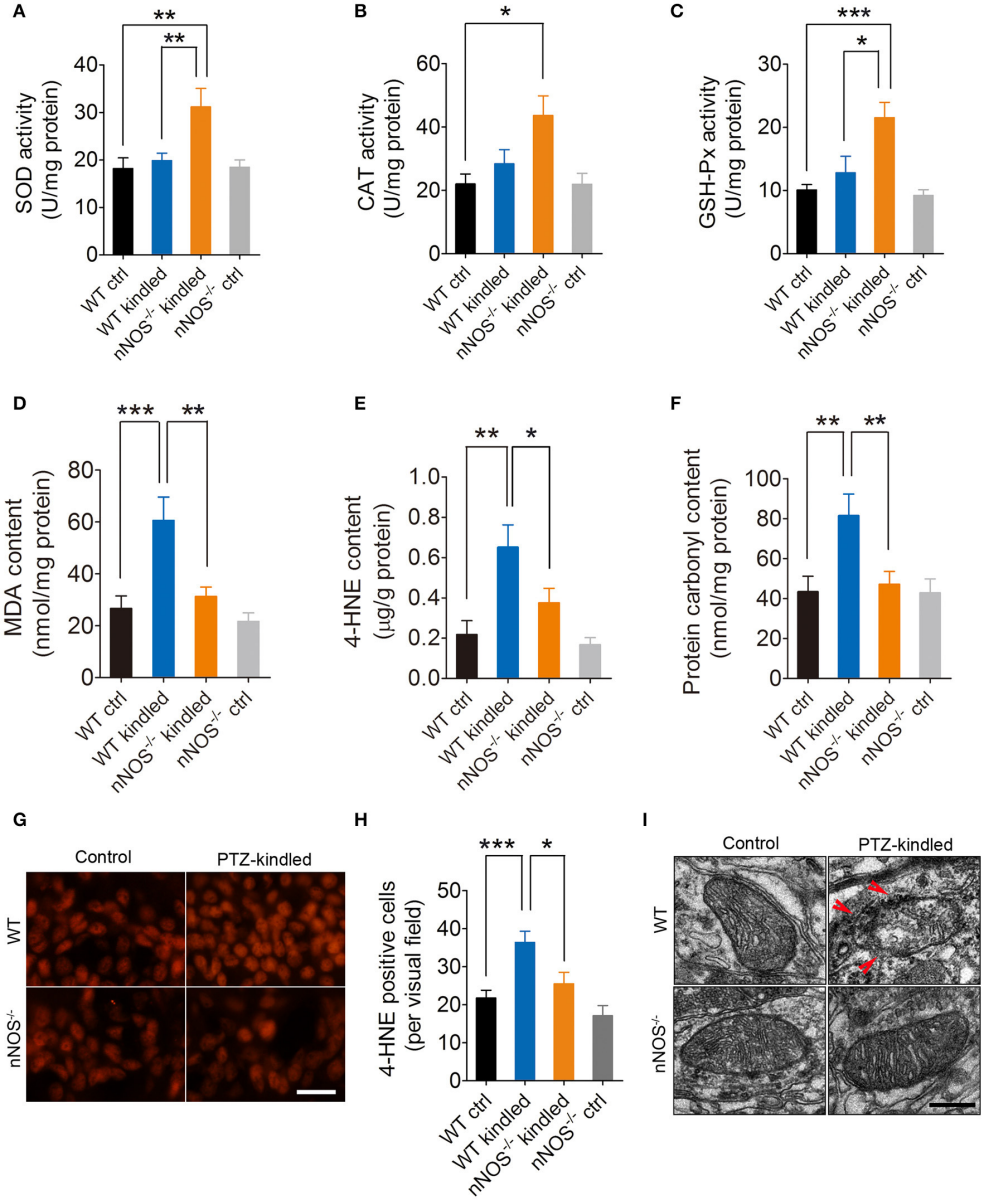
PTZ kindling induces hippocampal oxidative damage through activation of nNOS. **(A–C)** Bar graphs showing the quantification of the antioxidant enzymes of SOD, CAT, and GSH-Px in the hippocampus of WT ctrl, WT kindled, nNOS^−/−^ ctrl, and nNOS^−/−^ kindled mice (*n* = 5). For SOD activity, two-way ANOVA revealed a significant main effect of PTZ treatment [*F*_(1, 16)_ = 8.35, *p* = 0.011], genotype [*F*_(1, 16)_ = 5.40, *p* = 0.034], as well as PTZ treatment × genotype interaction [*F*_(1, 16)_ = 4.95, *p* = 0.041]. A Tukey *post-hoc* test revealed that nNOS^−/−^ kindled mice had significant higher SOD activity than WT ctrl mice (*p* = 0.002) and WT kindled mice (*p* = 0.006). For CAT activity, two-way ANOVA revealed a significant main effect of PTZ treatment [*F*_(1, 16)_ = 9.84, *p* = 0.006], but no significant effect of genotype [*F*_(1, 16)_ = 2.87, *p* = 0.11] nor PTZ treatment × genotype interaction [*F*_(1, 16)_ = 2.96, *p* = 0.105]. A Tukey *post-hoc* test revealed that nNOS^−/−^ kindled mice had higher CAT activity than WT ctrl mice (*p* = 0.04). For GSH-Px activity, two-way ANOVA revealed a significant main effect of PTZ treatment [*F*_(1, 16)_ = 15.65, *p* = 0.001] and PTZ treatment × genotype interaction [*F*_(1, 16)_ = 6.39, *p* = 0.022], but no significant effect of genotype [*F*_(1, 16)_ = 4.29, *p* = 0.055]. A Tukey *post-hoc* test revealed that nNOS^−/−^ kindled mice had higher GSH-Px activity than WT ctrl (*p* < 0.001) and WT kindled (*p* = 0.03) mice. **(D–F)** Bar graphs showing the quantification of the lipid peroxidation products of MDA and 4-HNE and protein oxidation indicator protein carbonyl in the hippocampus of WT ctrl, WT kindled, nNOS^−/−^ ctrl, and nNOS^−/−^ kindled mice (*n* = 5). For MDA content, two-way ANOVA revealed a significant main effect of PTZ treatment [*F*_(1, 16)_ = 14.38, *p* = 0.002], genotype [*F*_(1, 16)_ = 8.85, *p* < 0.009], as well as PTZ treatment × genotype interaction [*F*_(1, 16)_ = 4.74, *p* = 0.045]. A Tukey *post-hoc* test revealed that WT kindled mice had higher level of MDA content than WT ctrl mice (*p* < 0.001), and nNOS^−/−^ kindled mice had significant lower level of MDA than WT kindled mice (*p* = 0.002). For 4-HNE content, two-way ANOVA revealed a significant main effect of PTZ treatment [*F*_(1, 16)_ = 17.95, *p* < 0.001], genotype [*F*_(1, 16)_ = 4.66, *p* = 0.047], but no significant effect of PTZ treatment × genotype interaction [*F*_(1, 16)_ = 2.17, *p* = 0.16]. A Tukey *post-hoc* test revealed that WT kindled mice had higher level of 4-HNE than WT ctrl mice (*p* = 0.001), and nNOS^−/−^ kindled mice had significant lower level of 4-HNE than WT kindled mice (*p* = 0.021). For protein carbonyl content, two-way ANOVA revealed a significant main effect of PTZ treatment [*F*_(1, 16)_ = 6.79, *p* = 0.019], genotype [*F*_(1, 16)_ = 4.62, *p* = 0.047], but no significant effect of PTZ treatment × genotype interaction [*F*_(1, 16)_ = 4.32, *p* = 0.054]. A Tukey *post-hoc* test revealed that WT kindled mice had higher level of protein carbonyl than WT ctrl mice (*p* = 0.005), and nNOS^−/−^ kindled mice had significant lower level of protein carbonyl than WT kindled mice (*p* = 0.009). **(G)** Representative images of 4-HNE immunofluorescence in the hippocampus of WT ctrl, WT kindled, nNOS^−/−^ ctrl and nNOS^−/−^ kindled mice. **(H)** Bar graph showing the quantification of the 4-HNE immunopositive cells in the hippocampus of WT ctrl, WT kindled, nNOS^−/−^ ctrl, and nNOS^−/−^ kindled mice (*n* = 8). Two-way ANOVA revealed a significant main effect of PTZ treatment [*F*_(1,28)_ = 18.35, *p* < 0.001] and genotype [*F*_(1,28)_ = 8.34, *p* = 0.007], but no significant effect of PTZ treatment × genotype interaction [*F*_(1,28)_ = 1.36, *p* = 0.254]. A Tukey *post-hoc* test revealed that WT kindled mice had more 4-HNE positive cells than WT ctrl mice (*p* < 0.001), and nNOS^−/−^ kindled mice had fewer 4-HNE positive cells than WT kindled mice (*p* = 0.04). **(I)** Representative electron photomicrographs of the mitochondrial ultrastructure in the hippocampus of WT ctrl, WT kindled, nNOS^−/−^ ctrl, and nNOS^−/−^ kindled mice. Arrowheads indicate mitochondrial ultrastructure damage. Values are means ± S.E.M. ^*^p < 0.05, ^**^p < 0.01, ^***^p < 0.001, Two-way ANOVA, Scale bar=50 μm in **(G)** and 200 nm in **(I)**.

Redox homeostasis is important for maintenance of normal cellular activities. Alterations in redox homeostasis have been implicated in a number of neurological diseases (Sabens Liedhegner et al., 2012; Yin et al., 2014). During steady-state cellular conditions, cells have developed antioxidant systems to scavenge excessive free radicals to maintain redox homeostasis. Intracellular antioxidant enzymes, including superoxide dismutase (SOD), catalase (CAT), glutathione peroxidase (GSH-Px) are the most important antioxidant system. To compare the antioxidative abilities between PTZ-kindled and control mice and to determine whether the alteration of antioxidative abilities in PTZ-kindled mice is dependent on nNOS signaling, we detected hippocampal SOD, CAT, and GSH-Px enzymatic activities in nNOS^−/−^ mice and their wildtype littermates under normal and PTZ kindling conditions. Our results showed that the enzymatic activities of SOD, CAT, and GSH-Px were slightly increased in wildtype kindled mice compared to wildtype control mice. However, there is no significant difference (Figures 4A–C). The enzymatic activities of these antioxidant enzymes in nNOS^−/−^ kindled mice were significantly increased compared to wildtype control mice and wildtype kindled mice (Figures 4A–C), suggesting PTZ kindling impairs antioxidative abilities through activation of nNOS.

Excessive ROS/RNS-induced lipid peroxidation and protein oxidation are hallmarks of redox homeostasis impairment. Here, we found that the levels of peroxidation product MDA (Figure 4D) and 4-HNE (Figures 4E,G,H) in the hippocampus were both significantly increased in the wildtype mice after they are kindled, while in nNOS^−/−^ kindled mice, the levels of MDA (Figure 4D) and 4-HNE (Figures 4E,G,H) were significantly decreased compared to wildtype kindled mice. The production of MDA and 4-HNE in the hippocampus of nNOS^−/−^ and wildtype control mice remain similar (Figures 4D–E,G,H). Moreover, the protein oxidation indicator, protein carbonyl was also found increased in the hippocampus of wildtype kindled mice (Figure 4F), however, the content of protein carbonyl in the hippocampus of nNOS^−/−^ kindled mice were significantly decreased than that of wildtype kindled mice (Figure 4F). Protein carbonyl level in the hippocampus of nNOS^−/−^ and wildtype control mice remain similar (Figure 4F). These results suggest that PTZ kindling induces lipid peroxidation and protein oxidation through nNOS activation.

Increased oxidative stress was usually accompanied with mitochondrial dysfunction in epilepsy (Rowley et al., 2015). In the present study, we investigated the ultrastructure of the mitochondria in the hippocampal CA1 region by TEM in nNOS^−/−^ mice and their wildtype littermates under normal and PTZ kindling conditions. We found that the mitochondria in wildtype control mice were normal, and maintained intact inner and outer membranes, clear cristae and a smooth matrix, while in wildtype kindled mice, mitochondria displayed swelling, dilation, outer membrane ruptures, and cristae disruption (Figure 4I). These data are in agreement with our previous study (Zhu et al., 2016b). The mitochondria in nNOS^−/−^ kindled mice, however, maintained intact membranes, clear cristae and a smooth matrix, which were similar to those of wildtype control mice (Figure 4I), suggesting that the PTZ kindling-induced mitochondria damage is dependent on the activation of nNOS. Taken together, these data suggest that PTZ kindling provokes hippocampal oxidative stress through nNOS activation.

### PTZ kindling-induced hippocampal ER stress is dependent on nNOS activity

**Figure 5:**
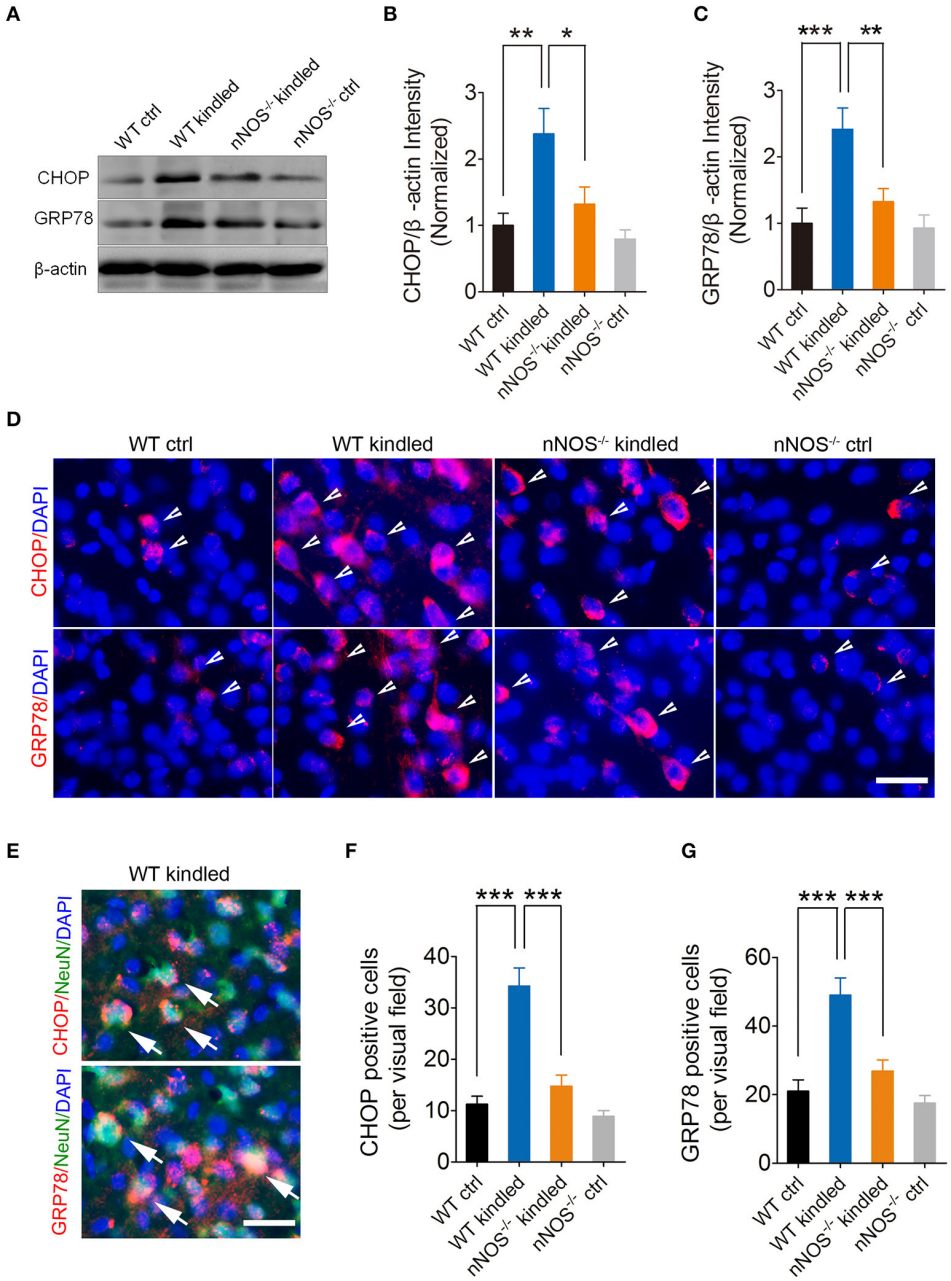
PTZ kindling-induced hippocampal ER stress is dependent on nNOS activity. **(A)** Western blot showing the protein levels of ER stress-related protein CHOP and GRP78 in WT ctrl, WT kindled, nNOS^−/−^ ctrl, and nNOS^−/−^ kindled mice. **(B,C)** Bar graphs showing the quantification of CHOP and GRP78 protein level in WT ctrl, WT kindled, nNOS^−/−^ ctrl, and nNOS^−/−^ kindled mice (*n* = **5)**. For CHOP, two-way ANOVA revealed a significant main effect of PTZ treatment [*F*_(1, 16)_ = 13.57, *p* = 0.002], genotype [*F*_(1, 16)_ = 5.98, *p* = 0.026], but not a significant effect of PTZ treatment × genotype interaction [*F*_(1, 16)_ = 2.72, *p* = 0.119]. A Tukey *post-hoc* test revealed that WT kindled mice showed a significant higher level of CHOP protein than WT ctrl mice (*p* = 0.002), and nNOS^−/−^ kindled mice showed a significant lower level of CHOP protein than WT kindled mice (*p* = 0.011). For GRP78, two-way ANOVA revealed a significant main effect of PTZ treatment [*F*_(1, 16)_ = 13.78, *p* = 0.002), genotype [*F*_(1, 16)_ = 5.51, *p* = 0.032], as well as a significant effect of PTZ treatment × genotype interaction [*F*_(1, 16)_ = 4.90, *p* = 0.042]. A Tukey *post-hoc* test revealed that WT kindled mice showed a significant higher level of GRP78 protein than WT ctrl mice (*p* < 0.001), and nNOS^−/−^ kindled mice showed a significant lower level of GRP78 protein than WT kindled mice (*p* = 0.005). **(D)** Representative images of the immunostaining of CHOP and GRP78 in the hippocampal CA1 region of WT ctrl, WT kindled, nNOS^−/−^ ctrl, and nNOS^−/−^ kindled mice respectively. The arrowheads indicate CHOP and GRP78 positive cells. **(E)** Representative images of CHOP or GRP78 (red) and NeuN (green) co-staining in the hippocampal CA1 region of WT-kindled mice. Nuclei were counterstained with DAPI (blue). The arrows indicate the CHOP/NeuN and GRP78/NeuN co-labeled cells. **(F–G)** Bar graphs showing the quantification of CHOP and GRP78 positive cells in WT ctrl, WT kindled, nNOS^−/−^ ctrl, and nNOS^−/−^ kindled mice (*n* = 8). For CHOP positive cells, two-way ANOVA revealed a significant main effect of PTZ treatment [*F*_(1, 28)_ = 39.79, *p* < 0.001), genotype [*F*_(1, 28)_ = 22.84, *p* < 0.001], as well as a significant effect of PTZ treatment × genotype interaction [*F*_(1, 28)_ = 13.99, *p* < 0.001]. A Tukey *post-hoc* test revealed that WT kindled mice showed significant more CHOP positive cells than WT ctrl mice (*p* < 0.001), and nNOS^−/−^ kindled mice showed significant fewer CHOP positive cells than WT kindled mice (*p* < 0.001). For GRP78 positive cells, two-way ANOVA revealed a significant main effect of PTZ treatment [*F*_(1, 28)_ = 27.04, *p* < 0.001], genotype [*F*_(1, 28)_ = 12.71, *p* = 0.001], as well as a significant effect of PTZ treatment × genotype interaction [*F*_(1, 28)_ = 6.72, *p* = 0.015]. A Tukey *post-hoc* test revealed that WT kindled mice showed significant more GRP78 positive cells than WT ctrl mice (*p* < 0.001), and nNOS^−/−^ kindled mice showed significant fewer GRP78 positive cells than WT kindled mice (*p* < 0.001). Values are means ± S.E.M. ^*^p < 0.05, ^**^p < 0.01, ^***^p < 0.001, Two-way ANOVA, Scale bar = 25 μm in **(D)** and 20 μm in **(E)**.

**Figure 6:**
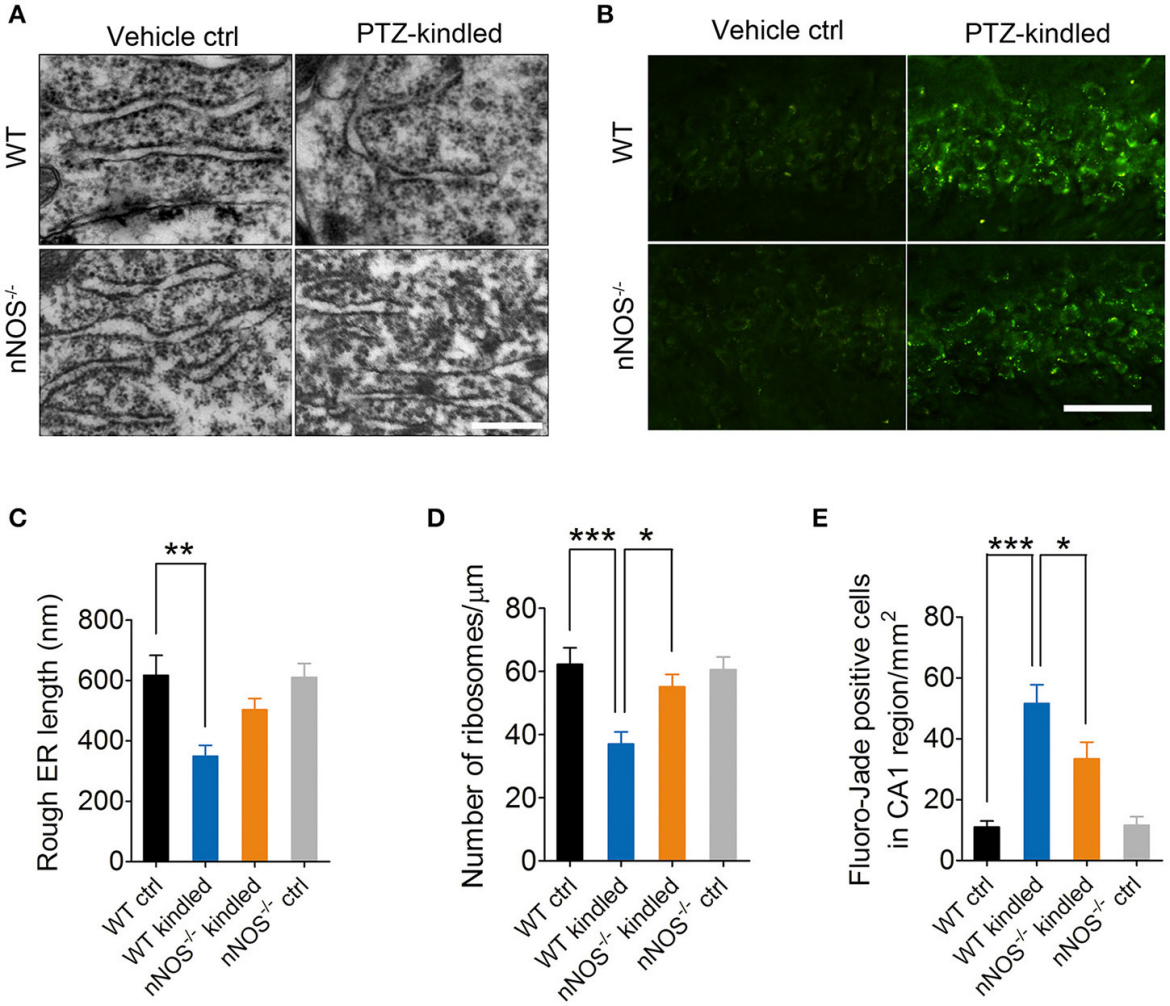
PTZ kindling induces hippocampal ER stress and neurodegeneration through activation of nNOS. **(A)** Representative electron photomicrographs of rough ER morphology in the hippocampal CA1 regions of WT ctrl, WT kindled, nNOS^−/−^ ctrl, and nNOS^−/−^ kindled mice. **(B)** Representative images of Fluoro-Jade B staining in the hippocampal CA1 regions of WT ctrl, WT kindled, nNOS^−/−^ ctrl, and nNOS^−/−^ kindled mice. **(C,D)** Bar graphs showing the quantification of length and ribosome density of rough ER (*n* = 12). For rough ER length, two-way ANOVA revealed a significant main effect of PTZ treatment [*F*_(1, 44)_ = 15.03, *p* < 0.001], but not a significant effect of genotype [*F*_(1, 44)_ = 2.29, *p* = 0.137], nor a significant effect of PTZ treatment × genotype interaction [*F*_(1, 44)_ = 2.73, *p* = 0.106]. A Tukey *post-hoc* test revealed that WT kindled mice showed a significant shorter rough ER length than that of WT ctrl mice (*p* = 0.008). For rough ER ribosome density, two-way ANOVA revealed a significant main effect of PTZ treatment [*F*_(1, 44)_ = 12.71, *p* < 0.001], genotype [*F*_(1, 44)_ = 3.62, *p* = 0.04], as well as a significant effect of PTZ treatment × genotype interaction [*F*_(1, 44)_ = 5.24, *p* = 0.027]. A Tukey *post-hoc* test revealed that WT kindled mice showed a significant lower ER ribosome density than that of WT ctrl mice (*p* < 0.001), and nNOS^−/−^ kindled mice showed a higher ER ribosome density than that of WT kindled mice (*p* = 0.032). **(E)** Bar graphs showing the quantification of Fluoro-Jade B positive cells in the hippocampal CA1 region (*n* = 5). Two-way ANOVA revealed a significant main effect of PTZ treatment [*F*_(1, 16)_ = 48.38, *p* < 0.001], genotype [*F*_(1, 16)_ = 3.85, *p* = 0.006], as well as a significant effect of PTZ treatment × genotype interaction [*F*_(1, 16)_ = 4.39, *p* = 0.025]. A Tukey *post-hoc* test revealed that WT kindled mice had more Fluoro-Jade B positive cells than WT ctrl mice (*p* < 0.0001), and nNOS^−/−^ kindled mice had fewer Fluoro-Jade B positive cells than WT kindled mice (*p* = 0.011). Values are means ± S.E.M. ^*^p < 0.05, ^**^p < 0.01, ^***^p < 0.001, Two-way ANOVA, Scale bar=500 nm in **(A)** and 25 μm in **(B)**.

Several lines of evidence demonstrated that nNOS-derived NO induces ER stress and consequently results in neuron death (Gotoh and Mori, 2006; Obukuro et al., 2013). To investigate whether nNOS is involved in PTZ kindling-induced ER stress, we firstly examined the ER stress markers, CHOP, and GRP78 by western blot in the hippocampus of wildtype and nNOS^−/−^ mice under control and PTZ-kindled conditions (Figure 5A). Our data revealed that the protein levels of CHOP and GRP78 in the hippocampus significantly increased in wildtype PTZ-kindled mice compared to wildtype control mice (Figures 5B,C). However, the protein levels of CHOP and GRP78 in the hippocampus of nNOS^−/−^ kindled mice significantly decreased compared to wildtype kindled mice (Figures 5B,C), suggesting nNOS deficiency suppressed PTZ kindling-induced ER stress level. The protein levels of CHOP and GRP78 in the hippocampus of nNOS^−/−^ control remain similar with those of wildtype control mice (Figures 5B,C). Secondly, we detected the expression of CHOP and GRP78 in the hippocampus of wildtype and nNOS^−/−^ mice under control and PTZ-kindled conditions by immunofluorescence. In consistent with the western blot data, our immunofluorescence results showed that hippocampal CHOP and GRP78 positive cells are significantly increased in wildtype kindled mice compared to wildtype control mice (Figures 5D,F,G). However, the CHOP and GRP78 positive cells in the hippocampus of nNOS^−/−^ kindled mice significantly decreased compared to wildtype kindled mice (Figures 5D,F,G). The CHOP and GRP78 positive cells in the hippocampus of nNOS^−/−^ control mice remain similar with those of wildtype control mice (Figures 5D,F,G). To further determine the cell type specificity of CHOP and GRP78 positive cells, we performed co-immunostaining using antibodies against CHOP/GRP78 and a neuronal marker, NeuN. Our results showed that ~67% of the CHOP-positive cells (Figure 5E, top) and 71% of the GRP78-positive cells (Figure 5E, bottom) in the hippocampus of PTZ-kindled mice were co-labeled with NeuN, indicating that the ER stress mostly occurred in neurons. To evaluate the rough ER morphology in the hippocampus of wildtype and nNOS^−/−^ mice under control and PTZ-kindled conditions, we performed TEM analysis on the hippocampal CA1 region of the mice. TEM studies demonstrated that rough ER cisternae were stacked with parallel arrays and there are large amount of ribosomes attached to ER in the hippocampal CA1 neurons of wildtype control mice (Figure 6A). In Wildtype PTZ-kindled mice, however, the compactness of ER stacks was disrupted, which causes the disorganization of rough ER (Figure 6A). Moreover, the rough ER length (Figure 6C) and the density of ribosomes attached to ER was also decreased (Figure 6D). However, the damage of ER in the hippocampus of nNOS^−/−^ kindled mice was attenuated compared to wildtype kindled mice (Figures 6A,C,D). Taken together, these data suggest that PTZ kindling induces hippocampus ER stress through activation nNOS.

### PTZ kindling-induced hippocampal neurodegeneration is dependent on nNOS activity

It is generally accepted that neurodegeneration is frequently occurred in epileptic condition. To investigate whether PTZ kindling-induced hippocampal neurodegeneration is dependent on nNOS activity, we examined the degenerating neurons by Fluoro-Jade B staining in the hippocampus CA1 region of wildtype and nNOS^−/−^ mice under control and PTZ-kindled conditions (Figure 6B). Our data revealed that the dying or degenerating neurons in the hippocampus significantly increased in wildtype PTZ-kindled mice compared to wildtype control mice (Figures 6B,E). However, the degenerating neurons in the hippocampus of nNOS^−/−^ kindled mice significantly decreased compared to wildtype kindled mice (Figures 6B,E), suggesting nNOS deficiency suppressed PTZ kindling-induced neurodegeneration. The degenerating neurons in the hippocampus of nNOS^−/−^ control remain similar with those of wildtype control mice (Figures 6B,E). Taken together, these data suggest that PTZ kindling induces hippocampus neurodegeneration is dependent on nNOS activity.

### PTZ kindling-induced oxidative damage and ER stress is dependent on nNOS-derived peroxynitrite

**Figure 7:**
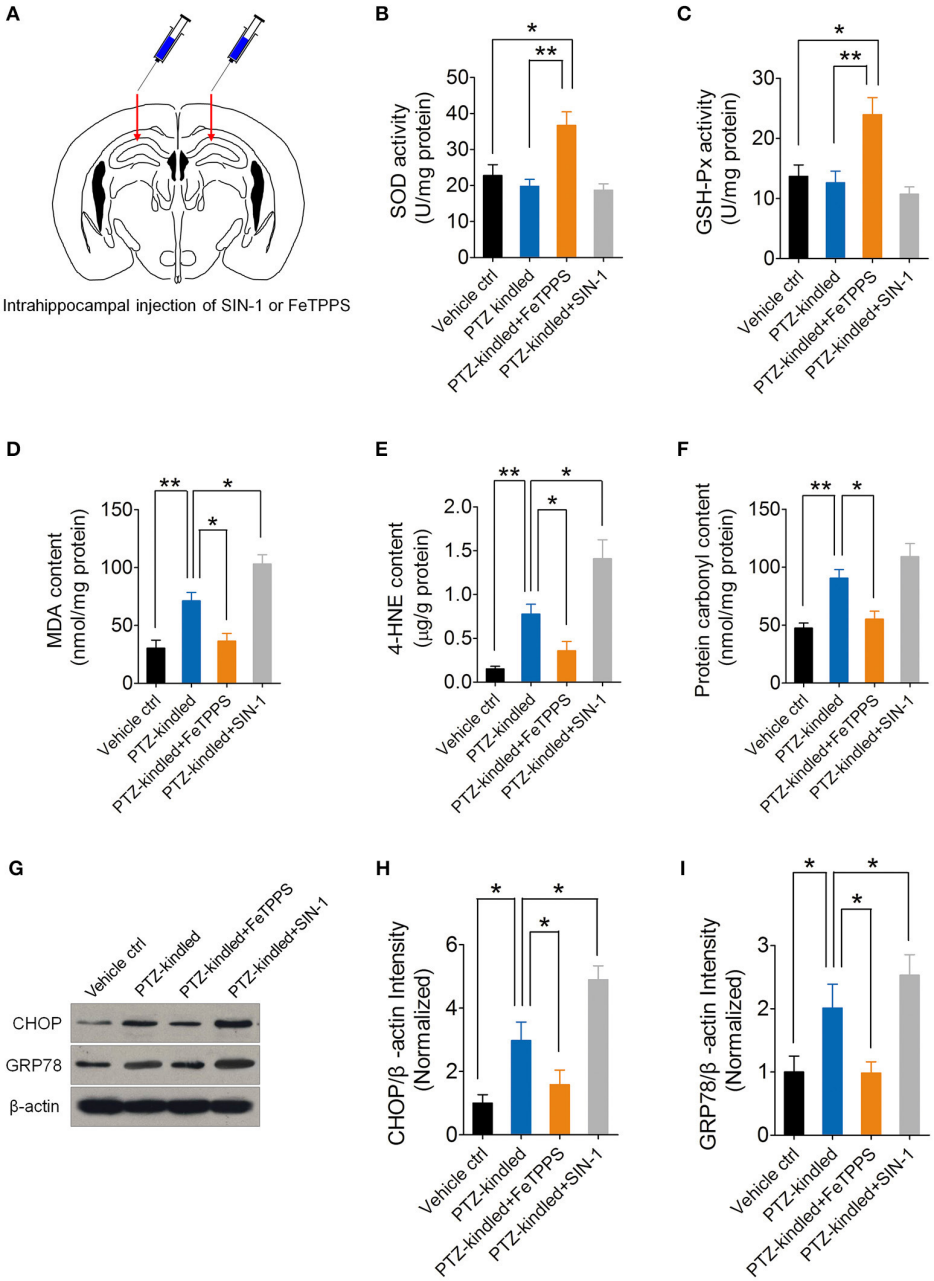
PTZ kindling-induced oxidative damage and ER stress is dependent on nNOS-derived peroxynitrite. **(A)** Schematic diagram of intrahippocampal microinjection of peroxynitrite donor SIN-I and peroxynitrite scavenger FeTPPS. Arrows indicate the hippocampal injection sites. **(B,C)** Bar graphs showing the quantifications of the antioxidant enzymes of SOD [*F*_(3, 16)_ = 9.16, *p* = 0.012, PTZ-kindled+FeTPPS vs. Vehicle ctrl; *p* = 0.003, PTZ-kindled+FeTPPS vs. PTZ-kindled] and GSH-Px [*F*_(3, 16)_ = 8.38, *p* = 0.013, PTZ-kindled+FeTPPS vs. Vehicle ctrl; *p* = 0.006, PTZ-kindled+FeTPPS vs. PTZ-kindled] in the hippocampus of Vehicle control (Vehicle ctrl), PTZ-kindled (PTZ-kindled), PTZ-kindled FeTPPS treated (PTZ-kindled+FeTPPS) and PTZ-kindled SIN-1 treated (PTZ-kindled+SIN-1) mice. (*n* = 5). **(D–F)** Bar graphs showing the quantifications of the lipid peroxidation products of MDA [*F*_(3, 16)_ = 22.10, *p* = 0.005, PTZ-kindled vs. Vehicle ctrl; *p* = 0.017, PTZ-kindled+FeTPPS vs. PTZ-kindled; *p* = 0.029, PTZ-kindled+SIN-1 vs. PTZ-kindled) and 4-HNE [*F*_(3, 16)_ = 17.25, *p* = 0.002, PTZ-kindled vs. Vehicle ctrl; *p* = 0.016, PTZ-kindled+FeTPPS vs. PTZ-kindled; *p* = 0.020, PTZ-kindled+SIN-1 vs. PTZ-kindled] and protein oxidation indicator protein carbonyl [*F*_(3, 16)_ = 13.82, *p* = 0.007, PTZ-kindled vs. Vehicle ctrl; *p* = 0.027, PTZ-kindled+FeTPPS vs. PTZ-kindled; *p* = 0.368, PTZ-kindled+SIN-1 vs. PTZ-kindled] in the hippocampus of Vehicle ctrl, PTZ-kindled, PTZ-kindled+FeTPPS and PTZ-kindled+SIN-1mice (*n* = 5). **(G)** Western blot showing the protein levels of ER stress-related protein CHOP and GRP78 in the hippocampus of Vehicle ctrl, PTZ-kindled, PTZ-kindled+FeTPPS and PTZ-kindled+SIN-1mice. **(H,I)** Bar graphs showing the quantification of CHOP [*F*_(3, 16)_ = 14.77, *p* = 0.032, PTZ-kindled vs. Vehicle ctrl; *p* = 0.027, PTZ-kindled+FeTPPS vs. PTZ-kindled; *p* = 0.016, PTZ-kindled+SIN-1 vs. PTZ-kindled] and GRP78 [*F*_(3, 16)_ = 6.87, *p* = 0.045, PTZ-kindled vs. Vehicle ctrl; *p* = 0.012, PTZ-kindled+FeTPPS vs. PTZ-kindled; *p* = 0.035, PTZ-kindled+SIN-1 vs. PTZ-kindled] protein levels in in Vehicle ctrl, PTZ-kindled, PTZ-kindled+FeTPPS and PTZ-kindled+SIN-1 mice (*n* = 5). Values are means ± S.E.M. ^*^p < 0.05, ^**^p < 0.01, One-way ANOVA.

nNOS-derived NO reacts rapidly with superoxide anion and produces peroxynitrite, which is a highly reactive nitrogen species that has been proved to have massive neurotoxicity. To further investigate whether peroxynitrite production is responsible for nNOS mediated ER stress and oxidative damage in the hippocampus of PTZ-kindled mice, we performed intrahippocampal injection of peroxynitrite donor SIN-1 and peroxynitrite scavenger FeTPPS in the PTZ-kindled mice (Figure 7A) and examined oxidative status and ER stress in the hippocampus. Firstly, we measured oxidative stress by detecting antioxidant enzymatic activities, lipid peroxidation and protein oxidation. Our results showed that peroxynitrite scavenger FeTPPS significantly increased the activities of antioxidant enzymes of SOD (Figure 7B) and GSH-Px (Figure 7C) and decreased PTZ kindling-induced lipid peroxidation products of MDA and 4HE (Figures 7D,E) and decreased PTZ kindling-induced protein oxidation indicator, protein carbonyl (Figure 7F). Peroxynitrite donor SIN-1 showed limited effects on the activities of SOD (Figure 7B) and GSH-Px (Figure 7C) in the hippocampus of PTZ-kindled mice, however, it aggravate the accumulation of lipid peroxidation products of MDA (Figure 7D) and 4HE (Figure 7E), suggesting peroxynitrite production is responsible for nNOS mediated oxidative damage in the hippocampus of PTZ-kindled mice.

Nest, we measure ER stress by detecting ER stress hall markers, CHOP and GRP78 with western blot (Figure 7G). Our results showed that peroxynitrite donor SIN-1 treatment significantly increased CHOP and GRP78 protein level in the hippocampus of PTZ-kindled mice (Figures 7H,I). However, peroxynitrite scavenger FeTPPS treatment significantly decreased CHOP and GRP78 protein level (Figures 7H,I), suggesting peroxynitrite production is responsible for nNOS mediated ER stress in the hippocampus of PTZ-kindled mice. Taken together, these data suggest that PTZ kindling-induced oxidative damage and ER stress is dependent on nNOS-derived peroxynitrite.

## Discussion

**Figure 8:**
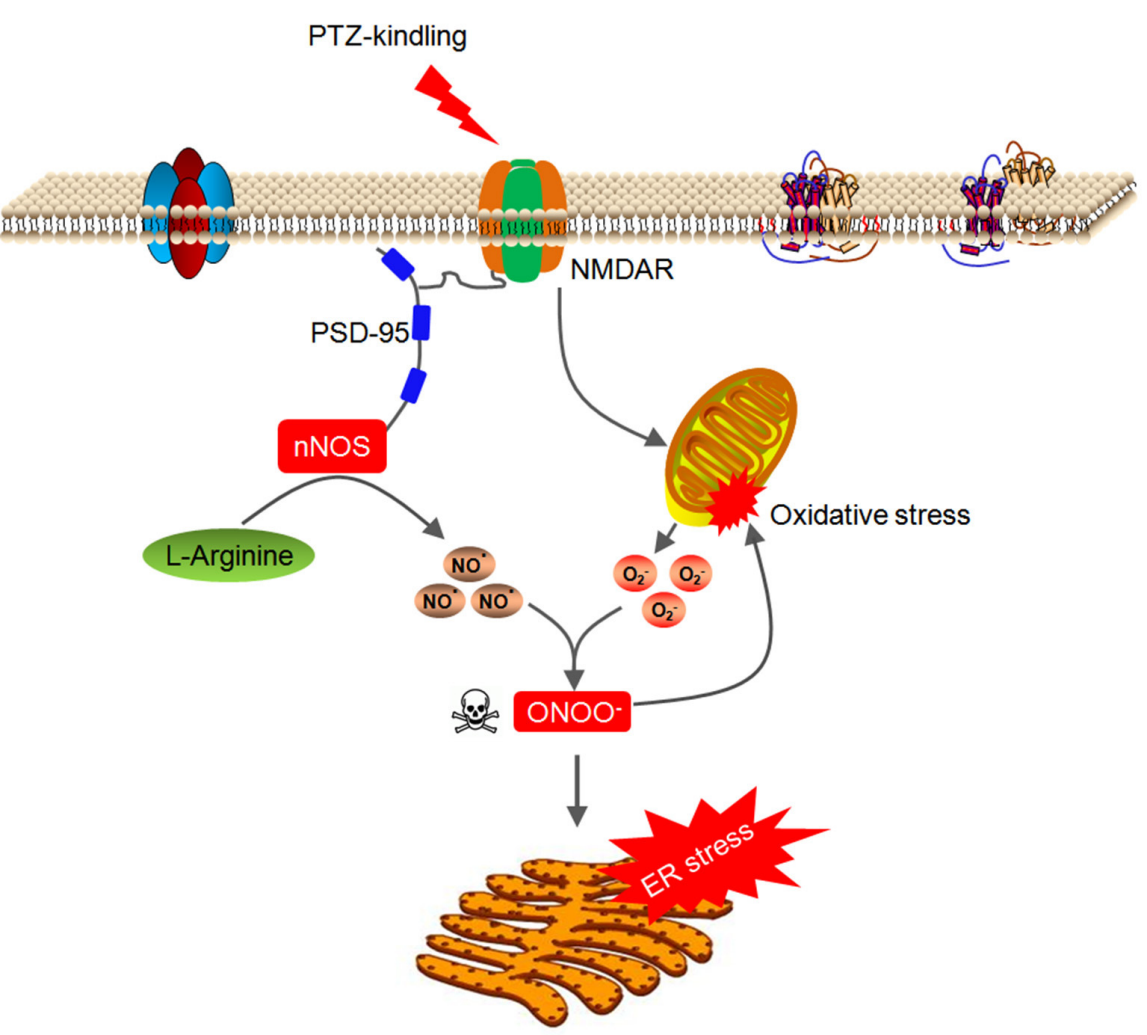
Model of signaling pathway whereby nNOS acts through peroxynitrite to trigger hippocampal ER stress and oxidative damage in PTZ-kindled epileptic conditions. PTZ kindling stimulates NMDA receptor which leads to nNOS activation and mitochondrial superoxide anion production. nNOS-derived NO rapidly reacts with superoxide anion and produces large amount of peroxynitrite. This PTZ kindling-induced peroxynitrite is neurotoxic and it disrupts redox homeostasis and damages the mitochondria and causes ER stress in the hippocampus.

In this study, we demonstrate that nNOS acts through peroxynitrite to trigger hippocampal ER stress and oxidative damage in the hippocampus of PTZ kindling mice. PTZ kindling stimulates NMDA receptor which leads to nNOS activation and mitochondrial superoxide anion production. nNOS-derived NO rapidly reacts with superoxide anion and produces large amount of peroxynitrite. This PTZ kindling-induced peroxynitrite is neurotoxic and it disrupts redox homeostasis and damages the mitochondria and causes ER stress in the hippocampus (Figure 8), which consequently contributes to neuronal injury in the pathophysiology of epileptogenic process.

The interactions between nNOS-derived NO and free radicals generated in seizures and other neurological conditions may cause severe oxidative stress (Rajasekaran, 2005; Hall et al., 2012). In the present study we found that large amount of superoxide anion was generated in the hippocampus of PTZ-kindled mice, and this newly generated superoxide anion rapidly reacts with nNOS-derived NO and produces plenty of peroxynitrite, which has been implicated in the pathology of various neurological disorders, including epilepsy (Chuang et al., 2009; Gonzalez-Reyes et al., 2016). Genetic deletion of nNOS abolished PTZ kindling-induced hippocampal peroxynitre production but not PTZ kindling-induced superoxide anion production, indicating that nNOS is necessary for PTZ kindling-induced peroxynitrite production in the PTZ kindling-induced epileptic condition.

Redox homeostasis is crucial for maintain normal functions of brain. To protect against the toxic effects of excessive ROS/RNS generated in the brain, antioxidant enzymes are activated to scavenge the free radicals. Here, we found that enzymatic activities of SOD, CAT, and GSH-Px were significantly increased in the hippocampus of nNOS^−/−^ kindled but not in wildtype kindled mice, indicating PTZ kindling disrupted redox homeostasis. In physiological state, endogenous antioxidant system in the hippocampus can clear up the normal production of ROS and RNS. However, in the pathological state of PTZ kindling epilepsy, the excessive production of ROS and RNS overwhelms the antioxidant system and causes oxidative damage. Depletion of nNOS, however, rescued antioxidant activities of PTZ-kindled mice. Lipid peroxidation and protein oxidation are hallmarks of redox homeostasis impairment. Our results showed that both lipid peroxidation and protein oxidation are increased in PTZ-kindled mice, however, depletion of nNOS suppressed PTZ kindling-induced lipid peroxidation and protein oxidation. Increased oxidative stress was usually accompanied with mitochondrial damage. Here we provided strong evidence that PTZ kindling severely damaged the hippocampal mitochondrial ultrastructure, however, depletion nNOS prevented this damage caused by PTZ kindling. Since abundant peroxynitrite was produced from the reaction of nitric oxide and superoxide anion during kindling process. Neither nitric oxide nor superoxide anion is a strong oxidant, however, peroxynitrite is a potent oxidant which can attack a variety of biological targets. Our findings thus raise the possibility that nNOS acts through peroxynitre to disrupt redox homeostasis and consequently leads to pathological damage in epilepsy disease.

Endoplasmic reticulum (ER) is responsible for synthesis and folding of secreted and membrane proteins. A variety of pathophysiological and environmental stimuli can disrupt ER homeostasis and proper ER functioning and consequently induce a pathological state known as ER stress (Roussel et al., 2013). Several lines of evidence suggest that ER stress is activated in epilepsy disease in both animal and human (Jang et al., 2004; Yamamoto et al., 2006; Chihara et al., 2011; Torres-Peraza et al., 2013). Consistent with these studies, here we observed significant ER stress in the hippocampus of PTZ-kindled mice, and this PTZ kindling-induced ER stress is dependent on nNOS activation. In the PTZ-kindled epileptic condition, NMDA receptor activation causes calcium influx and the generation of excessive NO from nNOS which is structurally linked to the NMDA receptor through PSD-95 (Bredt et al., 1991; Brenman et al., 1996; Nakamura et al., 2013). nNOS-derived NO rapidly reacts with PTZ kindling-induced superoxide anion and produces large amount of peroxynitrite. It has been reported that peroxynitrite causes ER stress in various cell types including human vascular endothelium cells (Dickhout et al., 2005) and neural stem cells (Lin et al., 2013). In agreement with these studies, here we found that peroxynitrite donor SIN-1 exacerbates PTZ kindling-induced ER stress while peroxynitrite scavenger FeTPPS attenuates PTZ kindling-induced ER stress, supporting the evidence that nNOS acts through peroxynitrite signaling to trigger hippocampal ER stress and oxidative damage in PTZ kindled mice. The mechanism by which peroxynitrite triggers ER stress in the epileptic condition remains to be determines. However, we speculate that excessive neuronal peroxynitrite might cause ER stress through mechanisms that involve S-nitrosylation of various protein targets. In support of this, Nakato et al. previously reported that S-nitrosylation of ER sensors IRE1α and PERK leads to dysfunctional ER stress signaling, thus contributing to neuronal damage (Nakato et al., 2015).

A growing body of evidence suggests that there is a direct link between ER stress and oxidative stress (Inoue and Suzuki-Karasaki, 2013; Kunchithapautham et al., 2014; Xu et al., 2015). Oxidative stress is caused by an imbalance between the production of free radicals and the ability to counteract the toxicity of these free radicals by antioxidant system. ROS is an important intracellular free radical responsible for the interaction between ER stress and oxidative stress. Overproduction of ROS in various pathological conditions targets ER calcium channels and causes ER calcium homeostasis disruption, leading to ER stress. On the other hand, a large amount of calcium release from the ER to the cytosol can stimulate mitochondria metabolism to produce more ROS (Zhang, 2010). Previous study has demonstrated ROS stimulates ER stress responses by activation of the unfolded-protein-response (UPR) pathway (Fedoroff, 2006; Rouault-Pierre et al., 2013). Conversely, ER stress triggers oxidative stress by stimulating ROS production and decreasing antioxidative enzymatic activities (Xu et al., 2005; Ozgur et al., 2014), suggesting a positive feedback of ER stress and oxidative stress relationship.

In summary, here we have used a PTZ kindling epilepsy model, supported by genetic nNOS deficient mice, to implicate nNOS as a pivotal signaling in PTZ kindling-induced ER stress and oxidative damage. Furthermore, we have demonstrated that nNOS acts through peroxynitrite to trigger hippocampal ER stress and oxidative damage in PTZ-kindled epileptic mice. Our understanding of the role of nNOS-peroxynitrite signaling in regulation of ER stress and oxidative damage in PTZ kindling-induced epileptic model may provide a therapeutic potential for neuroprotection in chronic epilepsy patients.

## Author contributions

XZ, JC, and HY designed research; XZ, JD, BH, RH, AZ, ZX, and HC performed research; XZ analyzed data and wrote the paper.

### Conflict of interest statement

The authors declare that the research was conducted in the absence of any commercial or financial relationships that could be construed as a potential conflict of interest.
